# The association between body mass index and efficacy of pembrolizumab as second‐line therapy in patients with recurrent/metastatic head and neck squamous cell carcinoma

**DOI:** 10.1002/cam4.5152

**Published:** 2022-08-17

**Authors:** Xinyi Zhang, Mengyu Rui, Chao Lin, Zhi Li, Dongliang Wei, Ruxue Han, Houyu Ju, Guoxin Ren

**Affiliations:** ^1^ School of Stomatology Weifang Medical University Weifang China; ^2^ Department of Oral Maxillofacial‐Head and Neck Oncology Ninth People's Hospital affiliated to Shanghai Jiao Tong University School of Medicine Shanghai China; ^3^ National Clinical Research Center of Stomatology Shanghai China

**Keywords:** body mass index, head and neck squamous cell carcinoma (HNSCC), obesity paradox, programmed cell death‐1 (PD‐1)

## Abstract

**Background:**

Recent evidence suggested a potential correlation between BMI and the efficacy of immune checkpoint inhibitors in cancer patients. This study aimed to evaluate the prognostic value of the body mass index (BMI) in recurrent/metastatic head and neck squamous cell carcinoma (R/M HNSCC) patients treat with pembrolizumab.

**Methods:**

The current retrospective cohort study enrolled 49 R/M HNSCC patients underwent at least one cycle of pembrolizumab as second‐line treatment from June 2018 to October 2020. Survival analysis of immunotherapy prognosis and risk factor analysis of age, gender, BMI, ECOG‐PS, CPS, rT‐stage, tumor site, and tube feeding.

**Results:**

Among the 49 patients, the BMI at the time of immunotherapy ranged from 14.5 to 32.0 kg/m^2^. The Kaplan–Meier analysis showed that the BMI was significantly correlated with overall survival time (OS, *p* = 0.0007) and progression‐free survival time (PFS, *p* = 0.0012). BMI, gender, prior treatment, serum albumin level, ECOG‐PS, CPS and rT‐stage were analyzed in multivariate Cox regression model analysis after adjusted for potential confounding clinical variables. Patients with underweight (OS:HR = 6.862, 95% CI:1.566–30.064, *p* = 0.011; PFS:HR = 5.672, 95% CI:1.364–23.586, *p* = 0.017);ECOG≥2 (OS:HR = 0.250, 95% CI:0.086–0.731, *p* = 0.011;PFS:HR = 0.284, 95% CI:0.101–0.805, *p* = 0.018); CPS <1(OS: HR = 4.34, 95% CI:1.271–15.464, *p* = 0.019; PFS:HR = 3.859, 95% CI:1.180–12.618, *p* = 0.025) and rT4‐stage(OS:HR = 4.380, 95% CI:1.452–13.209, *p* = 0.009;PFS: HR = 3.799, 95% CI:1.240–11.638, *p* = 0.019) suffered higher risk of mortality.

**Conclusions:**

The BMI at the time of clinical diagnosis was showed to be an independent predictive factor for R/M HNSCC patients receiving pembrolizumab. Compared with normal weight patients, underweight patients have worse clinical prognosis.

## Graphical abstract

BMI is independently associated with clinical outcomes in patients with R/M HNSCC. Compared to normal patients, underweight patients tend to mean higher risk of death. In survival analysis, overweight and obese patients have better OS and PFS than normal weight patients, and underweight patients had poorer prognosis.

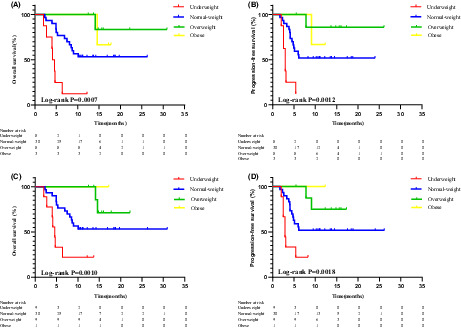

## 1 INTRODUCTION

Head and neck cancer (HNC), including malignancies involving the oral cavity, larynx, and pharynx, is the eighth most common cancer worldwide.^1^ Squamous cell carcinoma (SCC), which accounts for approximately 90.0% of HNCs, is known to recur or metastasize in 60.0% of patients with locally advanced HNSCC.^2^, ^3^, ^4^

Immunotherapy targeting programmed cell death 1 (PD‐1) and programmed cell death ligand 1 (PD‐L1) has emerged as a potential therapeutic option for recurrent or metastatic (R/M) HNSCC.^1^, ^2^ The KEYNOTE‐040 study (a randomized phase III trial that investigated the role of pembrolizumab vs. conventional treatment) showed that pembrolizumab, a humanized monoclonal antibody targeting PD‐1, prolonged median overall survival (OS) time and was therefore preferred over conventional treatment as a second‐line agent for treatment of R/M HNSCC.^3^ However, limited data are available regarding prognostic predictors and response to immunotherapy in patients with R/M HNSCC.

The tumor burden and treatment itself result in eating and swallowing difficulties in patients with R/M HNSCC, and undernutrition is commonly observed in this patient population (prevalence 42.0%–77.0%).^4^ In addition to oncological treatment, nutrition monitoring and support are indispensable, and early nutrition intervention is important to improve clinical outcomes of chemotherapy and radiotherapy (RT) in these patients.^5^ Undernutrition is often associated with severe toxicities, treatment interruptions, and poor survival.^6^, ^7^

The body mass index (BMI), a universal indicator of a patient's nutritional status, has gained much attention in oncology research recently.^8^, ^9^ High BMI is increasingly being recognized as a positive predictor of poor survival outcomes, following imj3munotherapy in several malignancies.^10^, ^11^, ^12^ Reportedly, high BMI has been associated with an increased risk of all‐grade immune‐related adverse events (irAEs).^13^ However, few studies have reported the association between BMI and prognosis and irAEs in patients administered immunotherapy for HNC.

In this study, using the Asian criterion of BMI, we retrospectively investigated the association between BMI and prognosis and irAE prevalence in patients with R/M HNSCC, who received pembrolizumab therapy as second‐line treatment.

## 2 MATERIALS AND METHODS

### 2.1 Clinical data and patient evaluation

The present study included 49 patients with HNSCC, who were treated at Shanghai Ninth People's Hospital between June 2018 and October 2020. The study was approved by the Institutional Ethics Committee of Shanghai Ninth People's Hospital, and all patients provided informed consent prior to study participation.

Patients' baseline clinical characteristics were recorded 1 day prior to the patient's first admission for immunotherapy. Treatment history was obtained from electronic medical records. Patients' height and weight were measured (with clothes and shoes), and the BMI was calculated using the following formula: BMI = kg/m^2^ (kg is the patient's weight measured in kilograms and m^2^ is the height in meters squared). The BMI cut‐off value for the Asian population is lower than that recommended by the World Health Organization (WHO) for the general (non‐Asian) population, which has been confirmed to be more suitable for the Asian population.^14^ Based on BMI cut‐off values, patients were categorized into the following subgroups: (obese: BMI ≥27.5 kg/m^2^, overweight: 23.5≤ BMI <27.5 kg/m^2^, normal weight: 18.5≤ BMI <23.5 kg/m^2^, and underweight: BMI <18.5 kg/m^2^). Tube feeding was defined as nutritional support administered through a nasogastric tube (NG) or percutaneous gastrostomy (PEG). The Eastern Cooperative Oncology Group Performance Status (ECOG‐PS) scores ranged from 0 (good performance status) to 5 (deceased).^15^ We defined irAEs as adverse events associated with PD‐1 immunotherapy, and these were graded according to the National Cancer Institute Common Toxicity Criteria for Adverse Events version 4.0.^16^ The combined positive score (CPS) was defined as the sum of PD‐L1 stained tumor cells and surrounding lymphocytes and macrophages divided by the total number of viable tumor cells multiplied by 100.^17^ We performed swimmer‐plot analysis (which is an early option to obtain a longitudinal response parameter) as recommended by the Prostate Cancer Clinical Trials Working Group 3.^18^

Inclusion criteria were as follows: (a) diagnosis of R/M HNSCC without indications for surgery or RT, (b) administration of at least one cycle of pembrolizumab mono‐immunotherapy, (c) administration of cetuximab and/or platinum‐containing therapy for R/M disease, with confirmed disease progression, (d) no prior PD‐1/PD‐L1 therapy, (e) no evidence of brain metastasis, and (f) availability of BMI data.

We assigned patients to a second‐line chemotherapy control group to determine whether our current findings were applicable to immunotherapy or any treatment modality selected in this patient population. The control group included 34 patients with HNSCC treated at Shanghai Ninth People's Hospital between April 2018 and November 2020. Inclusion criteria for the control group were the same as those used for the immunotherapy group except for “administration of at least one cycle of second‐line chemotherapy.” Evaluation criteria were the same as those outlined earlier.

### 2.2 Outcomes and evaluation

In this study, OS was established as the primary endpoint and the objective response rate, progression‐free survival (PFS), and toxicity as secondary endpoints. OS was defined as the interval between day one of pembrolizumab administration until death from any cause. Patients were censored at their last follow‐up visit if no event occurred. PFS was defined as the interval between day one of pembrolizumab administration and disease progression confirmed by investigator assessment or death from any cause. Treatment efficacy was evaluated every 6 weeks using magnetic resonance imaging or computed tomography based on Immune‐based Response Evaluation Criteria in Solid Tumors version 1.1.^19^ The imaging data were evaluated by two independent radiologists. Patients were followed up every 3 months for 1 year after treatment and every 6 months thereafter until death or data review, and the dates were randomly assigned to calculate survival time.

### 2.3 Statistical analysis

**FIGURE 1:**
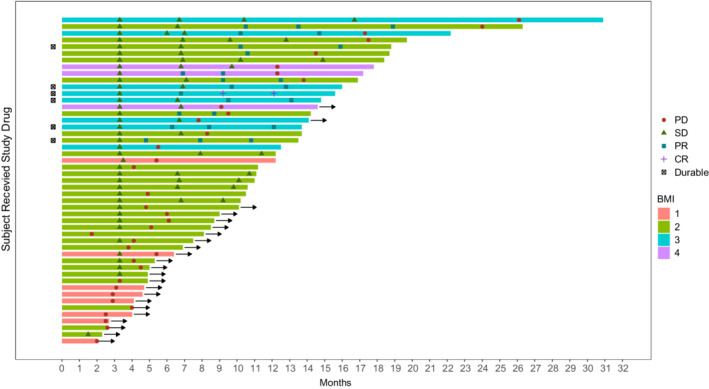
Swimmer‐Plot in 49 HNSCC patients who receiving immunotherapy. BMI1, underweight; BMI2, normal; BMI3, overweight; BMI4, obese; CR, complete response; PD, progressive disease; PR, partial response; SD, stable disease; Durable Responder is a subject who has confirmed response

The cut‐off time of our data was October 2021. The Fisher's exact test was used to analyze clinical data. The Cox regression model was used to determine confounders and effect modification of the parameters on the hazard ratio (HR) of the BMI (categorical variable) at the time of the patient's first admission for immunotherapy. Univariable Cox regression analysis was first used to confirm the association between the survival endpoint and each covariate. Covariates with *p* value <0.30 were subjected to multivariable analysis, and backward selection was used to establish the final multivariable model for PFS and OS. The Kaplan–Meier method was used to determine the distribution of PFS and OS in all patients and in subsets of patients with clinical benefit or primary resistance. The Chi‐square test was used to determine the association between irAEs and the BMI category. In the swimmer‐plot, each swim lane represents the beginning and end of each patient's immunotherapy treatment, and the different icons represent the clinical outcome assessment, with durable meaning that the immunotherapy treatment continues to be effective (Figure 1). Analysis of OS/PFS differences between immunotherapy and second‐line chemotherapy groups was tested for normal distribution. The Mann–Whitney U test was used in cases of non‐normal distribution, and the Fisher's exact test was used to confirm intergroup differences in BMI. A value of *p* < 0.05 was considered statistically significant. All data were analyzed using the SPSS software, version 26.0 (SPSS Inc.).

## 3 RESULTS

### 3.1 Clinical features of patients with head and neck squamous cell carcinoma

The study included 49 patients with R/M HNSCC (35 [71.4%] men and 14 [28.6%] women). Table 1 summarizes patients' clinical characteristics. Patients' median age was 59 years (range 34–79 years), and 23 (46.9%) patients were aged ≥60 years. The BMI ranged from 14.5 to 32.0 kg/m^2^; 3 (6.1%) patients were obese, 8 (16.3%) were overweight, 30 (61.2%) had normal weight, and 8 (16.3%) were underweight based on the WHO‐recommended BMI cutoff points for the Asian population. Tube feeding was performed in 19 (38.8%) patients, including in 5 patients with NG tube placement and in 14 patients who underwent gastrostomy. The serum albumin level ranged from 3.5 to 5.0 g/dl. ECOG‐PS scores ranged from 0 to 1 in 40 (81.6%) and were ≥2 in 9 (18.4%) patients. CPS was ≥1 in 23 (46.9%) and <1 in 26 (53.1%) patients. Recrudesce T stages (rT‐stage) were distributed as follows: T3 (22 [44.9%]) and T4 (27 [55.1%]). The most common primary tumor site was the tongue (20 [40.8%]), followed by the buccal mucosa (11 [22.4%]), the oropharynx (9 [18.4%]), the gingiva (3 [6.1%]), the palate (3 [6.1%]), the floor of the mouth (2 [4.1%]), and the maxillary sinus (1 [2.0%]). Analysis of clinical data using Fisher's exact test showed no differences between the BMI‐based subgroups with regard to some clinical characteristics (Table 1).

**TABLE 1:** Demographic and clinical data of 49 HNSCC patients

	Total (*N* = 49), No. (%)	Underweight (*N* = 8), No. (%)	Normal (*N* = 30), No. (%)	Overweight (*N* = 8), No. (%)	Obese (*N* = 3), No. (%)	*p*
Age (years)						
Median	59	58.5	59	61.5	57	0.999
Range	34–79	37–65	34–79	36–72	50–65	
<60	26 53.1	4 (50.0)	15 (50.0)	4 (50.0)	2 (66.7)	
≥60	23 46.9	4 (50.0)	15 (50.0)	4 (50.0)	1 (33.3)	
Gender						
Female	35 71.4	4 (50.0)	8 (26.7)	1 (12.5)	1 (33.3)	0.414
Male	14 28.6	4 (50.0)	22 (73.3)	7 (87.5)	2 (66.7)	
BMI						
Median	21.97	16.5	21.6	25.6	28.4	
Range	14.5–32.0	14.5–18.5	18.7–22.8	24.7–27.0	28.1–32.0	
ECOG‐PS						
0–1	40 (81.6)	3 (37.5)	26 (86.7)	8 (100.0)	3 (100.0)	0.068
≥2	9 (18.4)	5 (62.5)	4 (13.3)	0 (0.0)	0 (0.0)	
CPS						
<1	26 (53.1)	6 (75.0)	17 (56.7)	2 (25.0)	1 (33.3)	0.224
≥1	23 (46.9)	2 (25.0)	13 (43.30)		2 (66.7)	
rT‐stage						
T3	22 (44.9)	2 (25.0)	12 (40.0)	6 (75.0)	2 (33.3)	0.168
T4	27 (55.1)	6 (75.0)	18 (60.0)	2 (25.0)	1 (66.7)	
Tumor site						
Tongue	20 (40.8)	3 (37.5)	11 (36.7)	3 (37.5)	3 (100.0)	0.916
Buccal mucosa	11 (22.4)	3 (37.5)	6 (20.0)	2 (25.0)	0 (0.0)	
Oropharynx	9 (18.4)	1 (12.5)	5 (16.7)	3 (37.5)	0 (0.0)	
Gingival	3 (6.1)	0 (0.0)	3 (10.0)	0 (0.0)	0 (0.0)	
Palate	3 (6.1)	0 (0.0)	3 (10.0)	0 (0.0)	0 (0.0)	
Floor of mouth	2 (4.1)	1 (12.5)	1 (3.3)	0 (0.0)	0 (0.0)	
Maxillary sinus	1 (2.0)	0 (0.0)	1 (3.3)	0 (0.0)	0 (0.0)	
Tube feeding						
Yes	19 (38.8)	1 (12.5)	15 (50.0)	3 (37.5)	0 (0.0)	0.144
No	30 (61.2)	7 (87.5)	15 (50.0)	5 (62.5)	3 (100.0)	

Abbreviation: ECOG‐PS, Eastern Cooperative Oncology Group‐Performance Status.

### 3.2 Association between the body mass index and survival outcomes

The median follow‐up in our study was 11.1 months (range 3.0–31.3 months). Subgroup analysis based on BMI values showed that the 6‐month OS and PFS were 25.0% and 0.0% in underweight, 76.7% and 56.7% in normal‐weight, 100.0% and 100.0% in overweight, and 100.0% and 100.0% in obese patients, respectively. The 1‐year OS and PFS were 12.5% and 0.0% in underweight, 33.3% and 26.7% in normal‐weight, 100.0% and 75.0% in overweight, and 100.0% and 66.7% in obese patients. Comparison of 1‐year survival rates showed that survival outcomes were better in the overweight and in the obese than in the conventional group (comparison OS: 100.0%/100.0% vs. 33.3%; PFS: 75.0%/66.7% vs. 26.7%), and that the underweight group showed relatively poor prognosis (OS: 12.5% vs. 33.3%; PFS: 0.0% vs. 26.7%).

**FIGURE 2:**
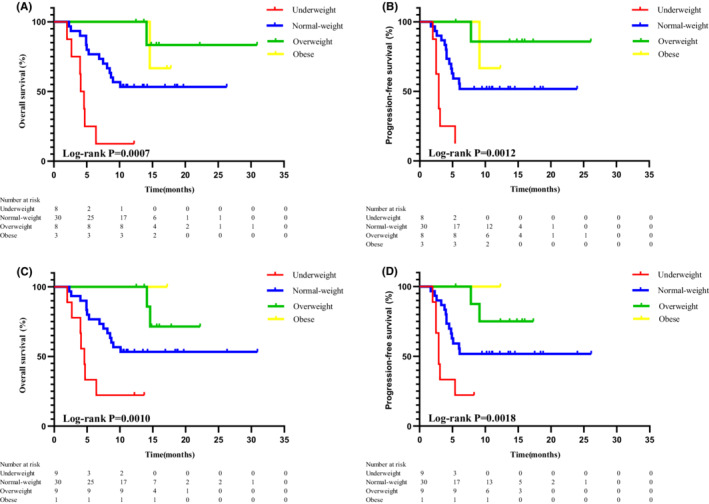
Patients receiving immunotherapy for head and neck cancer were grouped into underweight, normal weight, overweight and obese groups according to different criteria for survival analysis. (A) Asian standard overall survival; (B) Asian standard progression‐free survival; (C) WHO standard overall survival; (D) WHO standard progression‐free survival

The WHO criteria for the general population (non‐Asians) differ from those applicable to individuals of Asian descent. Underweight is defined as BMI <18.5 kg/m^2^, normal weight as 18.5≤ BMI <24.9 kg/m^2^, overweight as 25≤ BMI <29.9 kg/m^2^, and obesity as BMI ≥30.0 kg/m^2^ for the general population. Therefore, we recategorized our study population (49 patients with HNSCC) based on the WHO criteria applicable to the general population and performed Kaplan–Meier analysis. BMI was significantly correlated with OS and PFS (Asia‐OS/PFS: *p* = 0.0007/0.0012; WHO‐OS/PFS: *p* = 0.0010/0.0018) (Figure 2). OS and PFS were significantly associated with underweight (underweight vs. normal‐weight group, OS: hazard ratio [HR] 5.131, 95% confidence interval [CI] 1.959–13.443, *p* = 0.001; PFS: HR 4.690, 95% CI 1.776–12.385, *p* = 0.002) (Table 2). Following multivariate analysis after adjustment for potentially confounding clinical variables, the BMI remained an independent predictor of OS and PFS in patients with HNSCC; prognosis was poorer in the underweight than in the normal‐weight group (OS: HR 6.862, 95% CI 1.566–30.064, *p* = 0.011; PFS: HR 5.672, 95% CI 1.364–23.586, *p* = 0.017) (Table 3).

**TABLE 2:** Univariate analysis of clinical prognostic factors for clinical outcomes in 49 HNSCC patients

Variable	OS			PFS		
	HR	95% CI	*p*	HR	95% CI	*p*
BMI at diagnosis			**0.001**			**0.002**
Normal	Ref.					
Underweight	5.131	1.959–13.443	**0.001**	4.690	1.776–12.385	**0.002**
Overweight	0.181	0.024–1.391	0.100	0.194	0.025–1.479	0.114
Obese	0.438	0.056–3.401	0.430	0.489	0.064–3.737	0.491
Gender (vs. Female)	0.612	0.259–1.448	0.264	0.652	0.276–1.540	0.329
Age (vs.<60 years)	1.212	0.535–2.748	0.645	1.091	0.481–2.473	0.835
Prior treatment (vs. cetuximab)	2.739	1.013–7.410	**0.047**	3.280	1.205–8.931	**0.020**
Radiotherapy (vs. No)	0.635	0.261–1.546	0.317	0.535	0.219–1.307	0.170
Serum albumin level	0.945	0.861–1.037	0.230	0.940	0.857–1.031	0.191
ECOG‐PS(vs. ≥2)	0.168	0.068–0.412	**<0.001**	0.153	0.062–0.376	**<0.001**
CPS(vs. ≥1)	5.398	1.995–14.903	**0.001**	5.306	1.946–14.471	**0.001**
rT‐stage (vs. T3)	4.414	1.627–11.976	**0.004**	3.998	1.474–10.840	**0.006**
Tube feeding (vs. No)	1.342	0.587–3.071	0.486	1.404	0.614–3.208	0.421

*Note*: Underweight (BMI <18.5 kg/m^2^), Normal (18.5 ≤BMI <23.5 kg/m^2^), Overweight (23.5 ≤BMI <27.5 kg/m^2^), and Obese (BMI ≥27.5 kg/m^2^). OS, overall survival; PFS, progression‐free survival; CTX, cetuximab. CI, confidence interval; HR, hazard ratio; Ref, reference (HR = 1.0). Boldface indicates *p* < 0.05.

**TABLE 3:** Multivariate analysis of prognostic factors for clinical outcomes in 49 HNSCC patients

Variable	OS			PFS		
	HR	95% CI	*p*	HR	95% CI	*p*
BMI at diagnosis			**0.028**			**0.046**
Normal	Ref.					
Underweight	6.862	1.566–30.064	**0.011**	5.672	1.364–23.586	**0.017**
Overweight	0.229	0.028–1.887	0.171	0.260	0.032–2.135	0.210
Obese	0.947	0.089–10.047	0.964	0.863	0.091–8.156	0.898
Gender (vs. Female)	2.829	0.800–10.001	0.107	2.377	0.704–8.028	0.163
Prior treatment (vs. CTX)	1.368	0.385–4.860	0.628	2.439	0.732–8.1	0.147
Serum albumin level	1.120	0.990–1.268	0.073	1.104	0.981–1.243	0.102
ECOG‐PS(vs. ≥2)	0.250	0.086–0.731	**0.011**	0.284	0.101–0.805	**0.018**
CPS(vs. ≥1)	4.434	1.271–15.464	**0.019**	3.859	1.180–12.618	**0.025**
rT‐stage (vs.T3)	4.380	1.452–13.209	**0.009**	3.799	1.240–11.638	**0.019**

*Note*: CI, confidence interval; HR, hazard ratio; Ref, reference (HR = 1.0). Boldface indicates *p* < 0.05.

### 3.3 Association between other factors and clinical survival outcomes

Age, sex, prior systemic treatment, RT, serum albumin levels, tube feeding, ECOG‐PS scores, and CPS were subjected to univariate analysis. OS and PFS were significantly associated with prior treatment, ECOG‐PS scores, CPS, and the rT stage (Table 2). Multivariate analysis performed after adjustment for potentially confounding clinical variables showed that the ECOG‐PS scores, CPS, and the rT‐stage remained independent predictors of OS and PFS in patients with HNSCC, with an elevated mortality risk in those with ECOG ≥2 (OS: HR 0.250, 95% CI 0.086–0.731, *p* = 0.011; PFS: HR 0.284, 95% CI 0.101–0.805, *p* = 0.018), CPS <1 (OS: HR 4.34, 95% CI 1.271–15.464, *p* = 0.019; PFS: HR 3.859, 95% CI 1.180–12.618, *p* = 0.025), and rT4‐stage (OS: HR 4.380, 95% CI 1.452–13.209, *p* = 0.009; PFS: HR 3.799, 95% CI 1.240–11.638, *p* = 0.019) (Table 3).

### 3.4 Association between the body mass index and immune‐related adverse events

In this study, 8 (16.3%) patients developed any‐grade irAEs; 12.5%, 13.3%, 37.5%, and 0.0% of irAEs occurred in underweight, normal‐weight, overweight, and obese patients, respectively. However, high BMI was not significantly associated with irAEs in the current study (*p* = 0.375, Table 4). Table 5 shows the association between the incidence of irAEs and long‐term survival outcomes. We observed that irAEs were not significantly associated with better PFS (HR 0.730, 95% CI 0.217–2.460, *p* = 0.612) or OS (HR 0.653, 95% CI 0.194–2.198, *p* = 0.491).

**TABLE 4:** The analysis of the occurrence of any grade irAEs according to the BMI category

BMI‐patients (%)	irAEs of any grade		χ^2^ for trend
	Yes	No	
Underweight‐8 (16.3)	1 (12.5)	7 (87.5)	*p* = 0.375
Normal‐30 (61.2)	4 (13.3)	26 (86.7)	
Overweight‐8 (16.3)	3 (37.5)	5 (62.5)	
Obese‐3 (6.1)	0 (0)	3 (100)	

*Note*: irAEs, all the adverse events (AEs) that occurred during the immunotherapy.

**TABLE 5:** Univariate Cox proportional hazards regression for PFS and OS according to the occurrence of irAEs of any grade

Variable	irAEs of any grade (Yes versus No), HR (95% CI); *p* = value
PFS	0.730 (0.217–2.460); *p* = 0.612
OS	0.653 (0.194–2.198); *p* = 0.491

Abbreviations: CI, confidence interval; HR, hazard ratio; OS, overall survival; PFS, progression‐free survival.

### 3.5 Association between the body mass index and response to second‐line chemotherapy in patients with head and neck squamous cell carcinoma

Table 6 summarizes the clinical characteristics of 34 patients who received second‐line chemotherapy. We performed a normal distribution test for OS and PFS for second‐line chemotherapy vs. immunotherapy, both of which did not satisfy the normal distribution and were analyzed using the Mann–Whitney U test (OS: *p* = 0.048, Z = −1.977; PFS: *p* = 0.083, Z = −1.732). Fisher's exact test was used to analyze differences between the BMI‐based subgroups and showed that the intergroup differences in BMI were statistically nonsignificant (*p* = 0.999). BMI, sex, age, serum albumin levels, and ECOG‐PS scores were subjected to multivariate analysis. Mortality risk analysis showed that the remaining variables were statistically nonsignificant except for the ECOG‐PS score, which was an independent predictor of OS and PFS in patients with HNSCC, with an elevated mortality risk in those with ECOG ≥2 (vs. ECOG‐PS≥2, OS: HR 0.185, 95% CI 0.051–0.679, *p* = 0.011; PFS: HR 0.196, 95% CI: 0.052–0.740, *p* = 0.016) (Table 7).

## 4 DISCUSSION

Previous studies have reported that the nutritional status was significantly associated with and improved survival outcomes following PD‐1 inhibitor administration in patients with solid tumors.^20^ However, few studies have investigated the effects of nutritional status on outcomes of anti‐PD‐1 immunotherapy in patients with R/M HNSCC. We retrospectively investigated the association between BMI and clinical outcomes in patients with R/M HNSCC, who received pembrolizumab second‐line therapy. We observed that the BMI can be considered an independent prognostic factor, and patients with R/M HNSCC and concomitant overweight and obesity show higher OS and PFS than that observed in patients with R/M HNSCC and normal weight and that underweight patients tend to have shorter OS.

Patients with R/M HNSCC usually present with eating disorders that may lead to malnutrition and cachexia, which are strongly associated with poor prognosis.^21^ Overweight patients utilize their body's nutrient stores and can therefore achieve long‐term recovery with better prognosis.^22^ Cox regression analysis did not show an independent association between overweight or obesity and a reduced mortality risk; however, survival analysis showed that overweight and obesity were associated with favorable clinical outcomes, and survival rates were lower in underweight than in normal‐weight patients. Previous studies have reported that immunotherapy is significantly more effective in patients with a relatively high BMI (≥30 kg/m^2^) than in patients with normal BMI, with a two‐fold improvement in PFS and OS and >47.0% reduction in the mortality risk.^23^ These findings suggest that high BMI may be associated with favorable prognosis after administration of immunotherapy. These clinical data highlight significantly improved survival outcomes in obese patients with R/M HNSCC, who were administrated pembrolizumab, which suggests that patients with high BMI who receive immunotherapy may show longer survival time, lesser recurrence rates, and lower incidence of distant metastasis.^24^, ^25^ This phenomenon is referred to as the “obesity paradox,” which warrants further investigation.

Emerging evidence has shown that high BMI serves as a predictor of a high incidence of irAEs, which could be attributed to the fact that BMI may be associated with the activity and efficacy of PD‐1 immunotherapy.^26^ Unfortunately, we could not confirm these results owing to the relatively small sample size of this study and because we did not perform follow‐up for the results of each immunotherapy test (*p* = 0.375). Additionally, univariate Cox proportional hazards regression analysis did not confirm an association between irAEs and favorable PFS (*p* = 0.612) or OS (*p* = 0.491) in our study.

Notably, 30.0%–50.0% of patients with HNSCC are malnourished, and malnutrition is associated with high rates of postoperative complications; therefore, considering the poor response to treatment and high rates of tumor recurrence in this patient population, early nutritional intervention is important.^27^ Early nutritional interventions usually include administration of oral supplements or gastrointestinal tube feeding to compensate for inadequate oral intake.^28^ Based on Cox proportional hazards regression analysis, we did not observe an association between early nutritional intervention and mortality risk, which is attributable to the small sample size of the study. However, tolerance to follow‐up treatment, quality of life (QOL), and patient satisfaction were better in patients who underwent PEG/NG tube feeding than in those with a similar status at the same time. Patients who received tube feeding during treatment had lesser weight loss; however, no definitive conclusion can be drawn from this study.

Notably, serum albumin levels may reflect nutritional status and cancer treatment outcomes; changes in body cell mass and weight loss secondary to systemic inflammatory responses reduce serum albumin levels.^29^ However, in our study, the serum albumin level was not shown to be a risk factor that affected prognosis of R/M HNSCC.

HNC cells typically express the epidermal growth factor receptor (EGFR), which is associated with poor outcomes.^30^, ^31^ Cetuximab, an immunoglobulin‐G1 monoclonal antibody, inhibits the binding of the ligand to EGFR^32^ and also potentiates the activity of some chemotherapeutic agents, including cisplatin.^33^ Cetuximab is effective in cases of R/M HNSCC, and addition of cetuximab to cisplatin as first‐line therapy improves response rates compared with those associated with cisplatin monotherapy.^34^ However, owing to the small sample size of this study, multivariate analysis after adjustment of various factors did not conclusively establish that the mortality risk was lower in patients with R/M HNSCC treated with cetuximab. In addition to surgery and systemic therapy, RT is an important cornerstone of HNC treatment. In this study, we failed to establish an association between RT and clinical prognosis.

Food and Drug Administration approval of pembrolizumab monotherapy only in patients with CPS ≥1 represents the first mandated biomarker testing for selection of immunotherapy in HNSCC in the United States.^35^ CPS = 1 is increasingly being used in clinical practice as the cut‐off to predict prognosis. We observed that patients with CPS ≥1 had better prognosis in this study. Tumor T‐stage is also closely associated with prognosis based on tumor site and involved tissues. The rT‐stage was shown to be an independent predictor of mortality risk in our study, with a higher risk of death in patients with rT4.

Moreover, we obtained data on BMI and associated factors in patients treated with chemotherapy as second‐line treatment to verify whether our findings regarding the effects of BMI on survival were specifically attributable to immunotherapy. We did not observe an association between BMI and survival; however, we observed that ECOG‐PS scores ≥2 were independently associated with an elevated mortality risk. The ECOG‐PS, first described in 1982,^15^ is commonly used to assess the status of cancer treatment. PS is widely accepted as a predictor of important clinical outcomes, including QOL, chemotherapy toxicity, response to chemotherapy, terminal illness, PFS, and OS in patients with cancer.^36^ Previous studies have reported that ECOG‐PS scores may potentially affect the immune response.^37^ In our study, we observed that ECOG‐PS scores were significantly associated with the BMI in patients who underwent immunotherapy or chemotherapy and that patients with poor ECOG‐PS scores (≥2) showed unfavorable clinical prognosis and an increased mortality risk.

Following are the limitations of this study: (A) The small sample size and lack of data regarding anthropometric and nutritional markers (For example, kinetic or tomography guided adiposity measurements) are drawbacks. (B) We did not follow up changes in BMI in response to each immunotherapy treatment received, nor did we monitor changes in the QOL and psychological status of patients who underwent PEG/NG tube placement, using the EORTC core quality and QOL questionnaires. (C) The retrospective study design is also a limitation. Our findings should be validated in an independent cohort.

## 5 CONCLUSIONS

Kaplan–Meier analysis showed that OS and PFS were significantly better in obese and overweight patients with R/M HNSCC who received immunotherapy than in normal‐weight patients and that prognosis was poor in underweight patients. Underweight, ECOG‐PS scores ≥2, CPS <1, and rT4‐stage were independently associated with an increased mortality risk and may serve as independent prognostic indicators in patients with R/M HNSCC, who receive anti‐PD‐1 therapy. Further studies are warranted to definitively establish the association between overweight, obesity, and other variables and the mortality risk. Interestingly, we observed no statistically significant association between BMI and the mortality risk compared with controls among patients who received second‐line chemotherapy.

## AUTHOR CONTRIBUTIONS

Guoxin Ren and Houyu Ju supported the research. Xinyi Zhang, Zhi Li and Chao Lin collected the clinical data in our study. Xinyi Zhang performed statistical analysis. Dongliang Wei and Ruxue Han conducted the data analysis. Mengyu Rui wrote the manuscript.

## CONFLICT OF INTEREST

All the authors have no conflict of interest in this study.

## Supporting information


Appendix S1

## Data Availability

Relevant data in this study could be obtained from the corresponding authors by reasonable demand.
