# Supplementary material for: The association between body mass index and efficacy of pembrolizumab as second‐line therapy in patients with recurrent/metastatic head and neck squamous cell carcinoma
Source: Cancer Med. 2022 Aug 17;12(3):2702–12. doi: 10.1002/cam4.5152 (PMC9939135; doi:10.1002/cam4.5152)
Supplement: Supplementary file 1 — Appendix S1 [file CAM4-12-2702-s001.docx]

**Table 6**

Demographic and clinical data of 34 HNSCC patients.

|  | **Total**  (N=34)  NO. % | **Underweight**  (N=5)  NO.(%) | **Normal**  (N=21)  NO.(%) | **Overweight**  (N=6)  NO.(%) | **Obese**  (N=2)  NO.(%) | **P** |
| --- | --- | --- | --- | --- | --- | --- |
| **Age（years)**  Median  Range  ＜60  ≥60 | 61  37-74  16 47.1  18 52.9 | 57  37-62  4(50.0)  4(50.0) | 56  39-74  16(53.3）  14(46.7) | 57.5  42-68  4(50.0)  4(50.0) | 58  51-65  2(66.7)  1(33.3) | 0.770 |
| **Gender**  Female  Male | 17 50.0  17 50.0 | 2(40.0)  3(60.0) | 11(52.4)  10(47.6) | 4(66.7)  2(33.3) | 0(0.0)  2(100.0) | 0.822 |
| **BMI**  Median  Range | 22.3  16.6-31.1 | 17.4  16.6-18.5 | 20.9  18.8-23.3 | 26.1  23.8-27.1 | 29.5  27.9-31.1 |  |
| **ECOG-PS**  0-1  ≥2 | 22 64.7  12 35.3 | 3(60.0)  2(40.0) | 12(57.1)  9(42.9) | 5(83.3)  1(16.7) | 2(100.0)  0(0.0) | 0.186 |
| **Tumor site**  Tongue  Buccal mucosa  Gingival  Oropharynx  Palate  Floor of mouth | 16 47.1  9 26.5  5 14.7  2 5.9  1 2.9  1 2.9 | 2(40.0)  1(20.0)  1(20.0)  0(0.0)  0(0.0)  1(20.0) | 11(52.4)  6(28.6)  3(14.3)  1(4.8)  0(0.0)  0(0.0) | 2(33.3)  2(33.3)  1(33.3)  1(16.7)  0(0.0)  0(0.0) | 1(50.0)  0(0.0)  0(0.0)  0(0.0)  1(50.0)  0(0.0) | 0.368 |

Note: ECOG-PS, Eastern Cooperative Oncology Group-Performance Status.

**Table 7**

Multivariate analysis of prognostic factors for clinical outcomes in 34 HNSCC patients.

| **Variable** | **OS** |  |  | **PFS** |  |  |
| --- | --- | --- | --- | --- | --- | --- |
|  | HR | 95%CI | P | HR | 95%CI | P |
| **BMI at diagnosis** |  |  | 0.332 |  |  | 0.252 |
| Normal | Ref. |  |  |  |  |  |
| Underweight | 5.876 | 0.887-38.911 | 0.066 | 6.488 | 0.974-43.199 | 0.053 |
| Overweight | 1.632 | 0.281-9.483 | 0.585 | 1.378 | 0.211-8.987 | 0.738 |
| Obese | 1.529 | 0.135-17.376 | 0.732 | 4.605 | 0.352-60.204 | 0.244 |
| **Gender(vs.Female)** | 1.976 | 0.592-6.590 | 0.268 | 1.717 | 0.540-5.454 | 0.360 |
| **Age(vs.＜60years)** | 0.815 | 0.244-2.717 | 0.739 | 0.545 | 0.140-2.131 | 0.383 |
| **Serum albumin level** | 0.885 | 0.730-1.073 | 0.214 | 0.913 | 0.761-1.095 | 0.328 |
| **ECOG-PS(vs.≥2)** | 0.185 | 0.051-0.679 | **0.011** | 0.196 | 0.052-0.740 | **0.016** |

Note: CI, confidence interval; HR, hazard ratio; Ref, reference (HR=1.0). Boldface indicates P < 0.05.
